# Nitrous Oxide Gas

**Published:** 1864-09

**Authors:** G. R. Welding

**Affiliations:** Philadelphia


### NITROUS OXIDE GAS.

My attention has been drawn to this anæsthetic, by the publication of two articles in the *New York Dental Journal*, from different authors, who represent the extremes of opinion. Mr. W—— gives us a comprehensive transcript from the "books" and asserts the entire inadaptibility of any combination of nitrogen and oxygen to the purpose of producing insensibility to pain. His essay hardly rises above the dignity or thoughtfulness of an ordinary college thesis. He has proved nothing, and evidently knows nothing, from experience, of Medicine or Dentistry.

We dismiss Mr. W. with the request that he "walk the wards" a few months with some old physician, or earnest Dentist, before he write again about his old school-master theories. A man may grow very pedantic, or very dogmatic, at his desk; but it is rare that he becomes very *sensible.*

Mr. C. next appears in the ring. This man excites our terror. "Why," said a courtier to Napoleon, "do you send La Blanc into exile? He is only a fool," "I do not banish him," said Napoleon, "because he is a fool, I send him away because he is a *public* fool.

This Mr. C. compares to Mr. W. as a wooden man will compare with a thug. When Mr. W. states that his experience of six years as a teacher of chemistry, convince him beyond paradventure that nitrous oxide can never be rendered an auxilliary in surgery, we believe he is mistaken;—but when C. amiably smiles and says "I extracted over three thousand teeth, in three weeks, without pain," "it is as harmless as water," &c., &c., we know that C. is lying.

We dare not read C's article carefully. We are afraid to approach this mountain of mendacity, lest it fall on us and crush us. But, may we speak apologetically for this miserable man? Perhaps he inherited this vice. Some men are born with a predisposition to falsehood, just as some are to kleptomania. Such men are no more responsible for their thefts and lies than we are for having a large nose. It is only because a respectable journal has made its pages the means for the dissemination of such statements that we pause to correct them.

As we said; we dare not approach the moving mass of mighty lies marshalled into wordy order by this man. We content ourselves with making prisoners of such "stragglers," as may have dropped from the ranks.

"Anæsthesia was discovered by Dr. Horace Wells, of Hartford, Conn., on the 10th of December, 1844."

I can but suggest that this C. appoint himself literary executor to Horace Wells, and begin an action against one David Good, Strand, London, who published a book dated 1801, who wickedly gives the credit (due to C. and Horace Wells) to a swindling Englishman, named Sir Humphry Davy. See *Davy's Researches*, p. 87, *et seq.* An obscure pretender called John W. Webster, of Harvard, also mentions it in his lecture before his class in 1829. Webster suffered for his crimes, perhaps for the prospective robbery of the great and learned doctors and humanitarians, C. and Horace Wells. The late Dr. Horner used anæsthesia for the removal of *fungus hematodes*, while Professor of Anatomy in the University of Penn., about ten years before C., Wells & Co., had capacity enough to peddle tin ware, or shoe mules in New England.

Horace Wells drank, he committed suicide, but he never discovered.

God defend us from the sin of hard words to the *real* inventor. But fellows like C., Wells & Co., are not in that class. They haunt the doors of medical rooms, prowl about the doors of dentists, and thrust their ugly noses into the chemist's laboratory. They resemble that class of men called "body robbers" who steal upon the field of battle where brave men have died in fierce fight, to steal watches, and pick pockets.

Again C. says,—"It (nitrous oxide gas) is always safely used."

Alas!—

"Mortals rush where Angels fear to tread."

Many deaths have resulted from the use of this gas, Aneurism, in its protean varieties, is much two common to justify the operator in heedlessly rushing at an operation which may cost a valuable life. We believe that in all cases where the administration of this gas has been attended by fatal results, a faithful autopsy will show a preexisting arterial derangement.

We only object to the *general* use of this agent, because its proper administration requires more of the appliances of surgical and medical art, and, we may add, more knowledge than can be readily acquired by the mass of practitioners, who may be called upon at any moment to perform the smaller operations which properly belong to minor surgery.

Nitrous oxide gas, like amylene, ether, chloroform, &c., is placed in the family of anæthetics, that is, it produces insensibility to pain, by paralysing the nerves of sensation. But it is very unlike those agents in its manner of producing this insensibility. We believe ether, &c., produces paralysis, by depriving the blood of its usual supply of the life making element, oxygen. We are now satisfied that nitrous oxide gas, causes exhilerating obliviousness to operations, by forcing an *excess* of oxygen into the circulation.

It seems to be more electrical in its effects, producing less exhaustion on the nervous system, and to have a far less depressing effect on the ganglionic centers, than other agents.

Oxygen combines with nitrogen in five different proportions; three of these proportions form acide. Thus: Protoxide of nitrogen, binoxide of nitrogen, hypo-nitrous acid, nitrous acid, and nitric acid. Dr. Priestly mentioned it in 1772, and called it *dephlogisticated nitrous air*. The Dutch chemists called it *azote*. Davy relates that he breathed nine quarts of this laughing gas, for full three minutes! He further informs us that when nitrous oxide combines with water, the common air which was combined with the water is expelled. Dalton tells us that water absorbs nearly its own bulk of this gas. Hence if one insert a cylinder filled with this gas in a vessel of water (boiled), a great diminution of volume is seen. This absorption of the gas, and the evolution of common air by water is plainly caused by the superior affinity of water for the gas.

The process of manufacture is too familiar to be explained. We believe it to be *safer* and better adapted to Dentists' use than amalyne, ether, or chloroform. There is danger in any—*all* anæsthetics. For persons in sound health, it is safe, beyond question. But when auscultation, mediate, or immediate, shows any preexisting disease of the throacic viscera, or tendency to congestion in any vital part, its use is most clearly contra-indicated. For dissection, or removal of a tumor, or for the removal of a limb, no man of sense will commend it. Its duration is not sufficiently protracted. But (we repeat) for such minor operations as properly belong to the Dentist, it is a safe and steady friend.

Yours, truly,

G. R. WELDING.

*Philadelphia, July 29th.*

